# Thermal survival limits of larvae and adults of *Sirex noctilio* (Hymenoptera: Siricidae) in China

**DOI:** 10.1371/journal.pone.0218888

**Published:** 2019-06-26

**Authors:** Chengcheng Li, Lixiang Wang, Jiale Li, Chenglong Gao, Youqing Luo, Lili Ren

**Affiliations:** Sino-France Joint Laboratory for Invasive Forest Pests in Eurasia, School of Forestry, Beijing Forestry University, Beijing, China; Chinese Academy of Agricultural Sciences Institute of Plant Protection, CHINA

## Abstract

Temperature can be a major factor for the distribution of insects, especially among invasive insects. *Sirex noctilio* (Hymenoptera: Siricidae) has invaded many regions in China, causing enormous ecological and economic losses. We aimed to explore the trend and potential of diffusion by researching the thermal survival limits of *S*. *noctilio*. We measured the supercooling point (SCP), critical thermal temperature (CTmax), high lethal temperature (HLT) and low lethal temperature (LLT) for *S*. *noctilio* population in China and assessed life stage-related variation in thermal tolerance. Moreover, we determined the temperature tolerance range of *S*. *noctilio* and identified the temperature parameters for its potential invasive distribution risk analysis. The SCP of adults was -11.78 ± 0.67 (mean ± SEM), the CTmax was 37.67 ± 0.54, and those of larvae were -20.77 ± 0.44 and 40.53 ± 0.27, respectively. The LLT increased with exposure time, and the HLT was generally near 43°C. *S*. *noctilio* adults can tolerate higher temperatures than larvae, and the larvae showed high resistance to cold temperature. We calculated several temperature indexes based on our results, such as the lower temperature threshold (DV0) at -2.7°C, the upper temperature threshold (DV3) at 31°C, the temperature threshold for both heat stress (TTHS) at 35°C and cold stress (TTCS) at -32.5°C. We observed that, *S*. *noctilio* was not resistant to high temperatures, its CTmax is slightly lower than the lethal temperature, and the adults were more tolerant than larvae. Our next goal was to combine the temperature tolerance of symbiotic fungi, information on climate change and the current distribution of this species to predict its potential global distribution.

## Introduction

*Sirex noctilio* Fabricious (Hymenoptera: Siricidae) is an invasive woodboring wasp that attacks *Pinus* spp. (Pinaceae) in a wide range of habitats around the globe. It has invaded Australasia, North and South America, South Africa and China [1–6]. It has established populations in this regions of China [3]. The European woodwasp is native to Europe and northern Africa, where it is not an insect of economic importance, but it has become one of the most important pests in Southern Hemisphere forest plantations and has attracted much research to control its abundance [7–11].

In 2013, Li, Shi [3] discovered *S*. *noctilio* in northeastern China. In the field condition, we found that most of the Chinese populations of European woodwasps have one generation per year, while multiple generations occur in some years (Liu, unpublished data). The adults of *S*. *noctilio* emerge from early summer to early autumn [12]. Females lay their eggs after emergence, and the egg period is usually 16–28 days [13]. The optimal temperature for egg hatching is 25°C, and the lower temperature threshold for growth is 6.2°C [12]. A more recent study recalculated this lower threshold as 5.1°C [14]. *S*. *noctilio* has 6 to 12 larval instars, and requires temperatures between 12.5 and 33.5°C for complete development [12]. The diapause period occurs during the cold winter [mean temperature 3.7°C), and the overwintering larvae become dormant within the infested trunk (the temperature within an attacked tree is 1–5°C above the ambient temperature) [11]. The prepupal stage occurs as the temperature increases and lasts for approximately four weeks, while the pupal period is approximately 20–28 days induration; if the environment is sufficiently cool and humid, paralysis may occur in the second or third year after emergence [13, 15].

Temperature directly affects the growth, survival, geographical distribution and seasonal activity of insects [16, 17]. Therefore, the distribution or negative impact of invasive pests can also be affected by temperature [18]. The upper limit of heat resistance is often referred to as the critical thermal maximum (CTMax) and high lethal temperature (HLT), which can be detected by dynamic detection methods [19]. Investigating the low-temperature tolerance of insects usually adopts supercooling point (SCP) and low lethal temperature (LLT), which can reflect the lower limit of survival temperature of a species [20, 21]. Therefore, we aimed to identify the CTmax, SCP, HLT and LLT for *S*. *noctilio*.

Wood borers are unique in terms of their life stage variation and hidden habitat, such as *Anoplophora glabripennis* (Motschulsky) (Coleoptera: Cerambycidae), which has a dormant larval stage during the winter to avoid the adverse environmental conditions, has a wide distribution and causes severe damage to forests [22]. Woodborers are affected by temperature, similar to other insects with stage-based niche changes [23], and their tolerance of extreme temperatures are significantly varies among their different life stages.

In a previous study on the potential geographical range of *S*. *noctilio*, in Ireland, Bulman [10] identified the temperature thresholds of this species mainly according to data provided by Madden [12] and Nahrung [14]. The upper-temperature threshold for *S*. *noctilio* was also determined based on unpublished data regarding symbiotic fungi, while the temperature on which cold stress occurs was determined according to known *S*. *noctilio* distribution. We support the use of data on symbiotic fungi and the known distribution of *S*. *noctilio* to predict the potential areas of distribution for this species, however, the impact of some climatic factors, such as higher or colder temperatures, on *S*. *noctilio* as it colonizes new areas requires further research [11]. *S*. *noctilio* is an invasive species in China [3]. According to previous studies on genes and population sources, the populations in China have changed, with the current distribution differing from previous predictions; therefore, it is necessary to study the temperature tolerance of its Chinese populations [24].

Ours is the first of *S*. *noctilio* thermophysiology in China. We aimed to determine the temperature tolerance of *S*. *noctilio* on their different life stages in the laboratory and to identify and compare the temperature threshold of this species. We also analyzed the dispersal capacity of *S*. *noctilio* under these temperature thresholds. Moreover, we have discussed the mechanisms affecting temperature tolerance and distribution, the effects of climate change on *S*. *noctilio*, and specific parameters that can be used to predict climate-appropriate regions for this species.

## Materials and methods

### Sample collection

Our study had collected plant and animal from Pine plantation. Each experimental plots did not exit private ownership issues. Particular permission was not required since invasive species have exploded in plantations. Tested state-owned forest farms did not involve endangered or protected species.

*S*. *noctilio* larvae were collected from logs containing larvae in northeastern China from May 2017 to May 2018 (Table 1). All samples were taken from the sampling site approximately one week before the experiments. We fill the infested pines (weak *Pinus sylvestris* var. mongolica with resin droplets) and cut them into 1 m segments in the field. Then, it was wrapped in nets and moved to the quarantine laboratory (Beijing Forestry University) for temporary storage.

**Table 1:** Sampling location.

Locations	Geographic coordinates
Hegang City, Heilongjiang Province	47.33°N 103.27°E
Jinbao Town, Keerqin Left-wing Banner, Tongliao City, Inner Mongolia Autonomous Region	43.38°N 123.56°E
Yushu City, Jilin Province	44.83°N 126.55°E
Duerbert Mongolian Autonomous County, Daqing City, Heilongjiang Province	46.88°N 124.46°E

We used a wood splitter (LS7T-520, Shanghai Baiduan Industry and Trade Co., Ltd., China) to split the logs and collect larvae. The larvae collected during wintering period (October-November is recorded as the pre-wintering period, December-January is mid-wintering period, February-March is the post-wintering period) were for measuring the SCPs, among them, the larvae collected at mid-winter were performing the low-temperature-exposure experiments. The last-stage larvae were obtained in May to June and used for the critical high-temperature tests.

Before the emergence periods in 2017 and 2018, we also retrieved the damaged logs with a special cage as described above. During the emergence period (from June to September), we collected adults daily to measure the critical high temperatures and SCPs, and we also performed high-temperature-exposure experiments.

### CTmax measurement

The dynamic high-temperature method [25] was used to determine the critical high temperature for adults and mature larvae. The CTmax was mentioned by Cowtes and Bogert (1944), and it was defined as the temperature at which the body lost its ability to move after the wood-wasp had exhibited muscle twitching, increased frequency of activity, or ventral-facing position. The initial temperature for dynamic heating was set as 25°C, and the rate of temperature increase was set to 0.5°C/min (Ministat 230-cc-NR, Huber Ltd., Germany). More than 30 samples were used in each treatment.

### SCPs measurement

We measured the SCP of adults and late-stage larvae. The SCP of adults was measured as soon as the collected, while that of larvae was measured as early as they were removed from the wood. Thirty samples were evaluated per treatment. To conduct the measurements, we fixed the body of an individual to the thermistor probe of a four-way insect SCP test system (TP100, Jiangsu Senyi Economic Development Co., Ltd., China) with membrane and placed it in the test chamber (GDW-100, Beijing Yashilin Testing Equipment Co., Ltd., China) at -35°C. The temperature recorder documented the change in individual temperature (the cooling rate was approximately 1°C /min) until the body temperature increased suddenly increased, indicating that the fluid had entered a supercooled state. Each treatment was conducted in the same way. The temperature at which body fluid freezes is the freezing point (FP) and the moment at which latent heat is released during icing is the SCP.

### Thermal exposure experiment

We calculated adult mortality under high-temperature exposure and midwinter larval mortality under low-temperature exposure, to measure the thresholds for the long-term survival of *S*. *noctilio* at a constant temperature. High- and low-temperature incubators (GDW-100, Yiheng Scientific Instrument Co., Ltd., China) were used for the high-temperature-exposure experiments. The exposure experiments involved a gradient of 10 temperatures (25°C (control), 30°C, 33°C, 35°C, 37°C, 39°C, 40°C, 41°C, 43°C and 45°C) and 5 durations (2 h, 4 h, 8 h, 24 h and 48 h).

The low-temperature exposure experiments were performed with larvae in mid-winter [Li, unpublished results). Taking into account the actual cold temperatures, we identified the lower threshold using a gradient of eight temperatures (5°C, 0°C, -5°C, -10°C, -15°C, -20°C, -25°C and -30°C) for 2 h, 4 h, 24 h and 48 h.

After the treatments were completed, the samples were placed in a standard environment (25°C, dark: light = 24: 0 h, relative humidity = 75%) for 2 h and touched the body of each larva with a probe to determine whether it was alive. Three to five larval replicates were used in each treatment, and each treatment was repeated three times. The semi-lethal temperature (LLT_50_, which results in 50% mortality) and the complete lethal temperature (LLT_99_, i.e., 99% mortality) were also determined.

### Statistical analyses

To determine the lethal temperatures (50% and 99%), the probability model (and its 95% confidence interval) was fitted to the survival data using the SPSS statistical software version 23.0 (probit; SPSS Inc., Cary, NC). Differences in temperature tolerance between adults and larvae were analyzed with t-test, and differences acquisition periods were analyzed with Fisher's LSD test (SPSS 23.0). Simple descriptive statistics calculations (mean ± SEM), linear model analysis and the identification of the frequency distribution of the CTmax were conducted with GraphPad Prism version 7 (GraphPad Software Inc., La Jolla, CA, USA).

## Results

### Critical thermal maximum of adults and last-instar larvae

**Fig 1:**
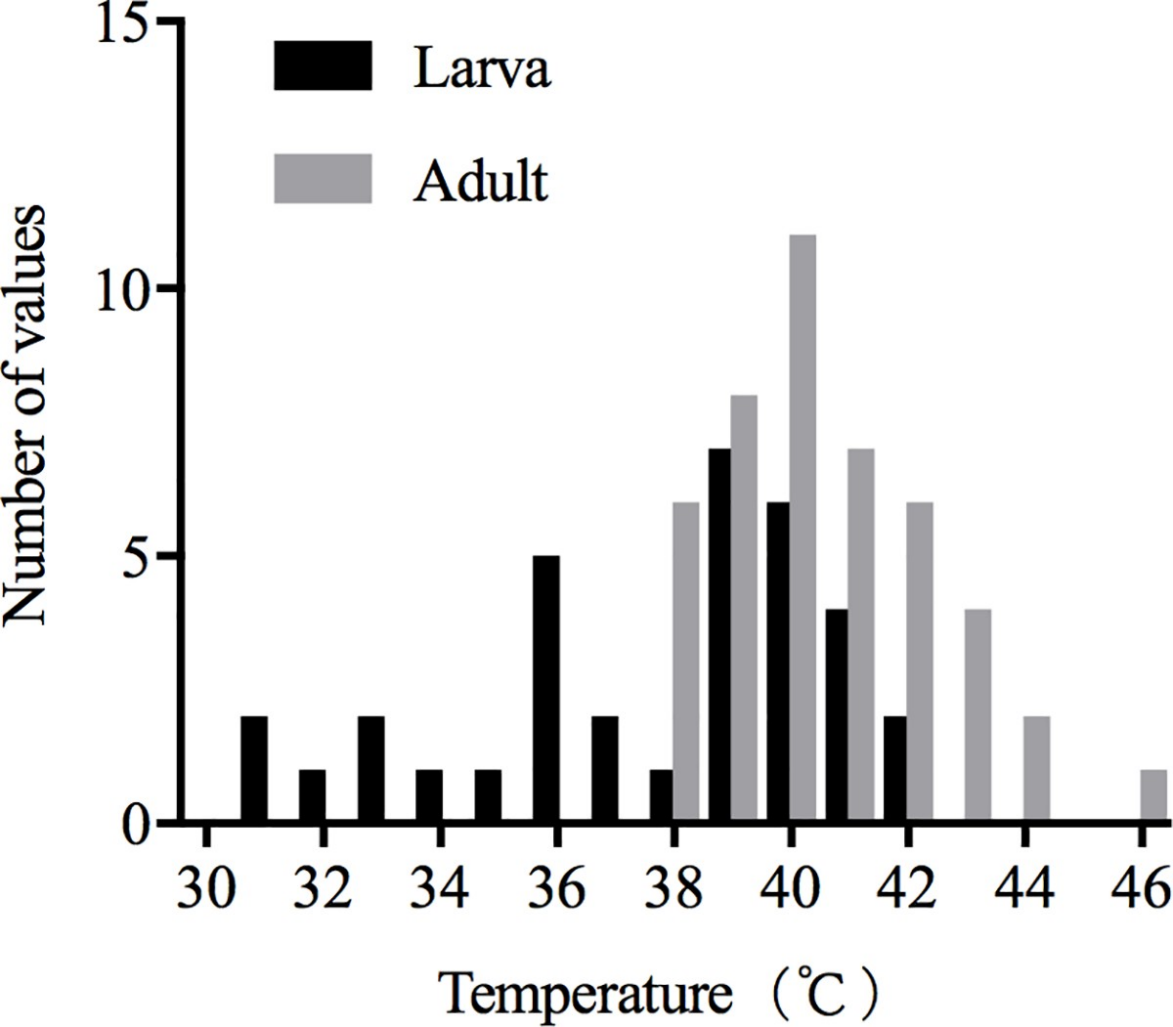
Frequency distribution of CTmax of *S*. *noctilio*. The grey column represents the number of adults died between the range on the X Axis, and the black column represents the same content of the larva (P < 0.0001 in Mann-Whitney test).

**Fig 2:**
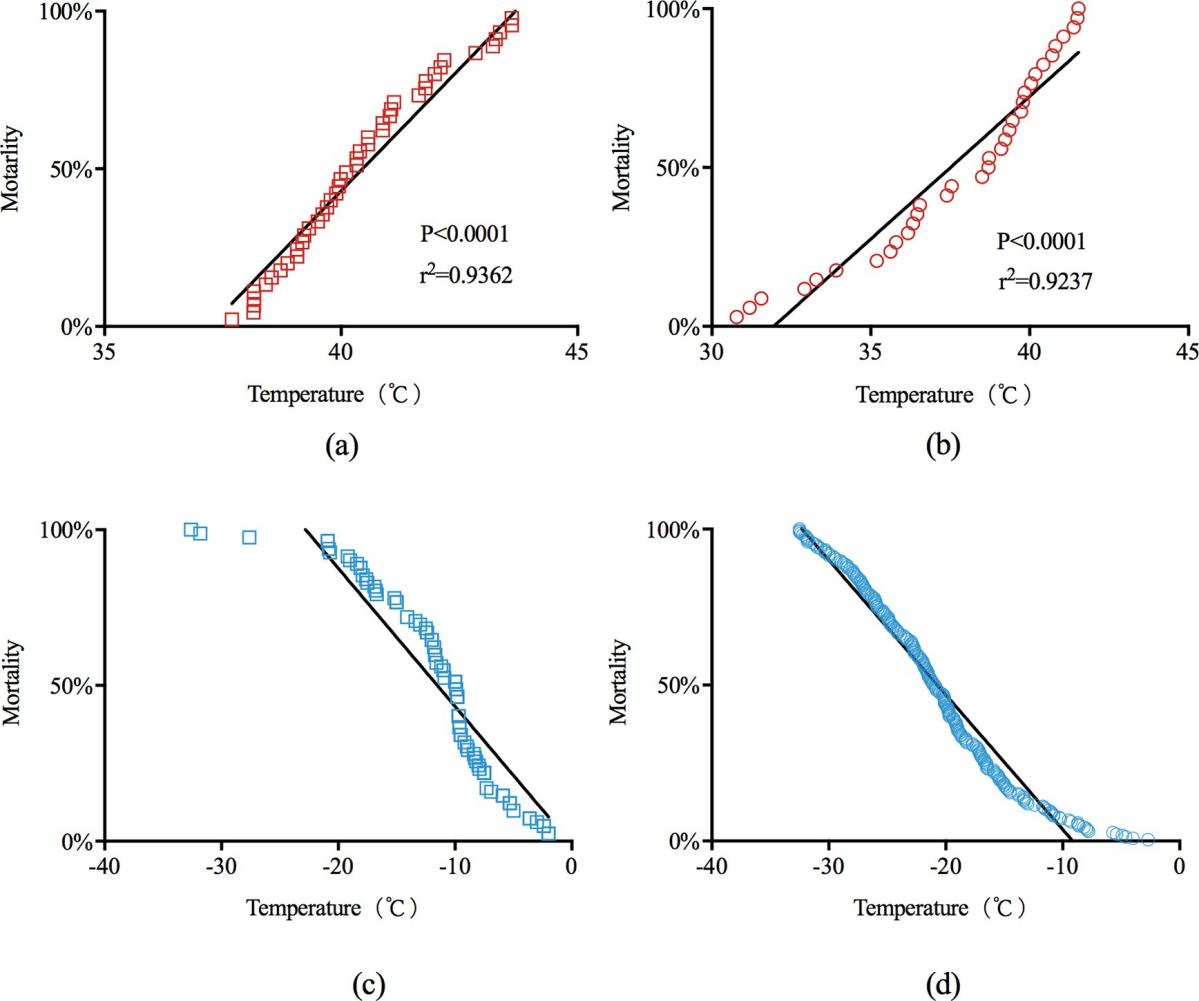
Straight line fitting graph of mortality with temperature rising. Since the experimental observation found that the individual was in a state of death after the experiment, the Y-axis used “mortality”. (a) CTmax of adults (y = 0.1546x - 5.754, F = 631.3, d.f. = 1;43). (b) CTmax of larvae (y = 0.08982x - 2.869, F = 387.3, d.f. = 1;32). (c) SCP of adults (y = -0.04436x - 0.01056, F = 457, d.f. = 1;80). (d) SCP of larvae (y = -0.04319x - 0.3949, F = 5480, d.f. = 1;319).

The comparison of the critical temperatures of larvae (37.67 ± 0.54°C, n = 34) and adults (40.53 ± 0.27°C, n = 45) showed that the adults were higher resistant to heat (t = 5.08, d.f. = 77, P < 0.0001) (Fig 1). As shown in the figure, the critical high temperature of adult insects ranged from 37.69°C to 45.84°C, which means that high temperatures below 37.69°C uncaused adult heat shock, and when the temperature rose to 45.84°C and above, would causes all adults enter the coma and shock state. The corresponding temperature range for larvae was 30.78°C to 41.54°C. The critical high temperature of most individuals (adults and larvae) was between 39 and 41°C, so this is the main temperature range that threatens *S*. *noctilio* with heat shock, and could also be regarded as an important inflection point of *S*. *noctilio* resistance to high-temperature stress. In addition, the mortality of the adults and larvae at different temperatures were calculated, and the HLT_99_ values (mortality > 99%) were 42.53°C (y = 0.1546x - 5.5754, r^2^ = 0.9362, F = 631.3, d.f. = 1;43, P < 0.0001) (Fig 2A) and 43.08°C (y = 0.08982x - 2.869, r^2^ = 0.9237, F = 387.3, d.f. = 1;32, P < 0.0001) (Fig 2B), respectively. Thus, the CTmax of *S*. *noctilio* adults was higher than that of larvae.

### Supercooling point of adults and larvae

**Fig 3:**
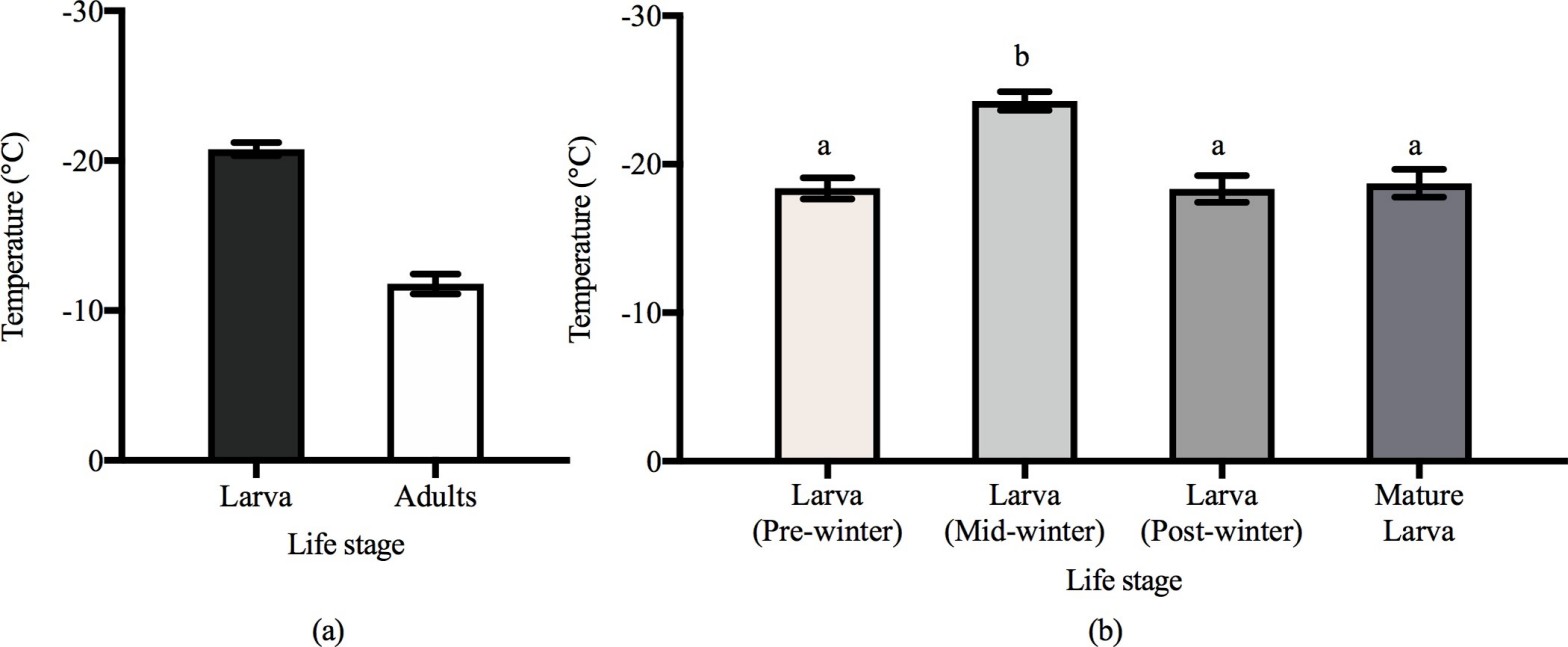
SCP (mean ± SEM) for different life stages of *S*. *noctilio*. (a) The SCP of all larval samples and adults. (b) The SCP of larvae that collecting from different periods, pre-winter means Oct. 2017 to Nov.2017, min-winter means Dec. 2017 to Jan. 2018, post-winter means Feb. 2018 to Mar. 2018 and late-stage larvae were collected during May. 2017 to Jun. 2017. The alphabet means that there is a difference P < 0.01 in Fisher's LSD test.

We found that the overall SCPs of the larvae (-20.77 ± 0.4378, n = 349) were lower than those of the adults (-11.78 ± 0.665, n = 82) (t = 10.85, d.f. = 305, P < 0.0001) (Figs 2C, 2D and 3A). We measured different overwinter larval SCPs (range: -32.5 to -2.7°C) for the pre-winter (-18.37 ± 0.71, n = 106), mid-winter (-24.27 ± 0.62, n = 111), and post-winter state (-18.42 ± 0.73, n = 102), and we observed significant differences in these overwintering periods (F = 26.616, d.f. = 2; 319, P < 0.01) (Fig 3B). To eliminate the possible effects of ambient temperature, we compared the SCP of emerged adults and late-stage larvae (-18.72 ± 0.94, n = 30) collected at almost the same time, with the results also showing a lower larval SCP (t = 4.042, d.f. = 93, P < 0.0001).

### Lethal temperature of mid-wintering larva and adult

**Fig 4:**
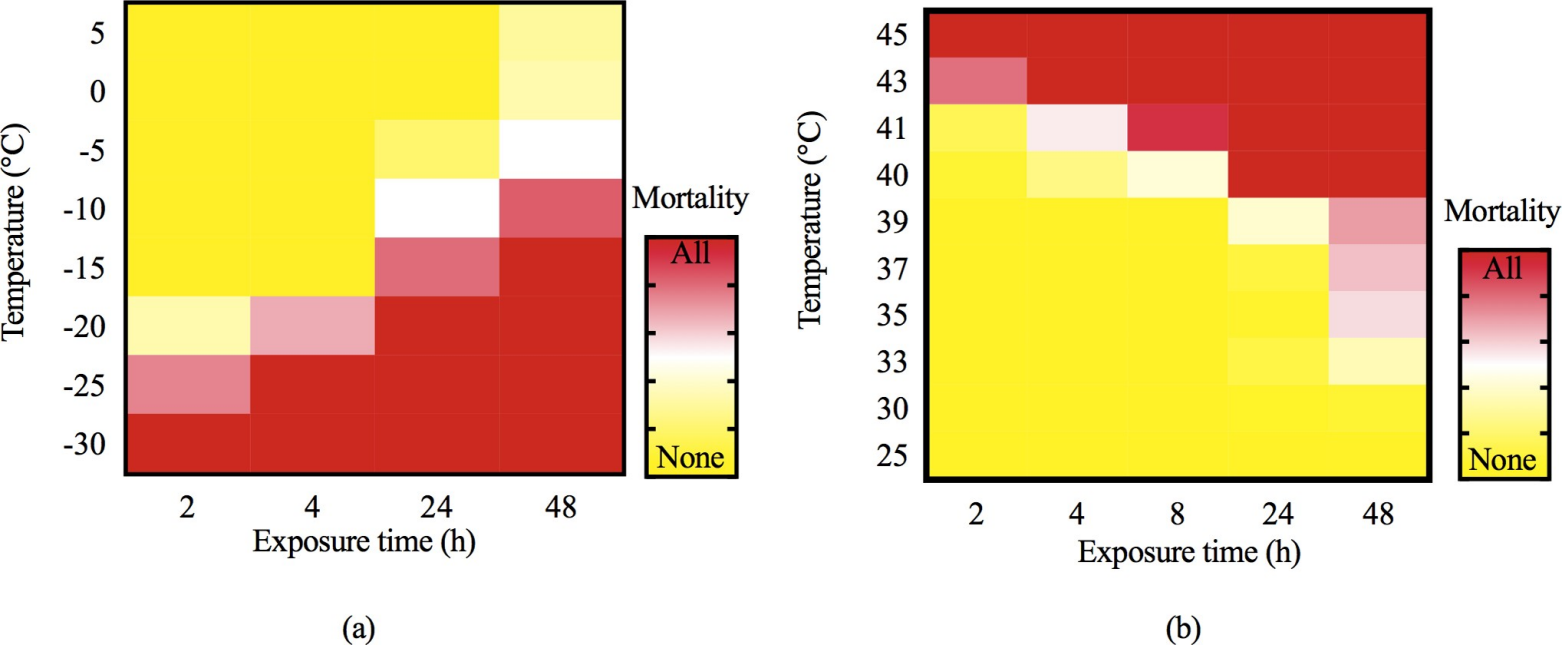
The statistical heat maps of mortality under different temperature for various exposure duration. The gradual transition from yellow to red corresponds to an increase in the mortality of samples from none to all. (a) Mortality of larvae at low low-temperature exposure. (b) Mortality of adults at high high-temperature exposure.

The low lethal temperature of the overwintering larvae gradually increased with an increase of exposure duration, and the trend of thermal lethality exhibited a similar pattern (Fig 4). Short-term exposure (< 8 h) to -20 and 41°C resulted in death. Furthermore, the lethal temperature was very close to the standard temperatures after prolonged continuous exposure (> 8 h), and these were -15 and 40°C.

Probabilistic analysis of response variables showed that LLT_50_ and LLT_99_ gradually increased, HLT_50_ gradually decreased, and HLT_99_ was stable at 42.95°C (±0.35) (Table 2). The wintering larvae of *S*. *noctilio* were able to tolerate short-term extreme hypothermia, but cannot tolerate long-term low temperature stress.

**Table 2:** Lethal temperature of *S*. *noctilio* under different exposure times.

Exposure time (h)	slope + SE	LLT_50_ (°C)	95% fiducial limit		LLT_99_ (°C)	95% fiducial limit		chi-square
			Lower	Upper		Lower	Upper	
Cold exposure of larvae								
2	-0.30+0.09	-22.09	-24.522	-19.75	-29.77	-39.79	-26.56	0.67
4	-0.41+0.14	-19.23	-21.453	-16.94	-24.84	-35.79	-22.25	0.56
24	-0.21+0.05	-10.00	-12.488	-7.51	-21.07	-30.17	-17.21	0.83
48	-0.16+0.04	-3.53	-6.25	-0.41	-17.89	-28.57	-13.28	0.92
Thermal exposure of adults								
2	0.63+0.10	42.03	41.45	42.74	45.70	44.56	47.81	0.97
4	1.35+0.34	40.52	40.21	41.01	42.25	41.54	44.24	1.00
24	0.455+0.06	38.12	36.63	39.86	43.23	41.00	50.41	0.00
48	0.27+0.03	34.87	33.96	35.68	43.38	41.70	46.06	0.26

## Discussion

Temperature limitations affecting the survival of *S*. *noctilio* have not been addressed in previous studies. Here, we aimed to analyze the thermal physiology of this species and its variation its different life stages. We chose the life stages of larvae and adults, as they experience extreme temperatures and have significantly different habitats, allowing the exploration of temperature tolerance [11, 21, 26].

### The high-temperature threshold of *S*. *noctilio* was revealed by its CTmax and lethal high temperature

The comparison of the lethal temperatures of adults and larvae during dynamic warming [27] indicates that *S*. *noctilio* adults exhibit stronger heat resistance than larva during the same period. The reasons for this result are as follows: 1) Life history: *S*. *noctilio* emerges when the temperature is relatively warm (summer to early autumn), and high temperatures would speed up its emergence [11, 15]. 2) Habitat: the larvae live in the tunnels located in the xylem of the host, while adults have direct contact with the atmosphere. Furthermore, Goulet, Ryan [11] found the differences between the internal and external air temperatures of the infested trunk by *S*. *noctilio*. Besides, although the larvae can eat pure symbiotic fungi under laboratory conditions [28], they cannot be artificially reared in the absence of logs, and hunger or environmental changes during the experiment might result in decrease of larval tolerance [29].

According to the results of the high-temperature-exposure experiments with adults, the HLT_50_ increased with the exposure time, while the HLT_99_ (>2 h) varied by approximately 43°C, which was near the critical maximum temperature of adults (42.53°C) (Fig 2). At the same time, 43°C was the lower limit temperature in the exposure experiment that caused adult death over a short period (2 h) (Fig 3B). Therefore, we think that this is the critical temperature that affects the short-term survival of *S*. *noctilio*. The high temperature may affect the expression and response of heat shock proteins [30] and even alter the breathing pattern of insects [31], which requires further investigation. The HLT_50_ at 48 h (34.87°C) for adults was approximately 3°C lower than the minimum critical temperature (37.69°C). It indicated that the lethal high temperature of adults would decrease with the prolongation of stress time.

The adults of *S*. *noctilio* has large differences in body size, and it might be necessary to analyze the relationship between body size characteristic values (such as weight) and cold tolerance. It could be possible to obtain a similar result as “the bigger individuals of *Solenopsis invicta* (Hymenoptera: Formicidae) are more susceptible to the effects of cold environments [32]”.

### The low-temperature threshold of *S*. *noctilio* revealed by SCP and low lethal temperature

Referring to Fig 3 and the linear regression (Fig 2C and 2D), the cold tolerance of adults was lower than that of larvae, which is consistent with the life history of this species: *S*. *noctilio* survives in the larval state at the tunnel of the host xylem during winter [1, 11, 12]. The larvae could only survive for a short period after leaving the xylem, so the longest treatment duration was only 48 h in the exposure experiment. The methods used in the experiment for adults were based on Ma and Ma [23]. The conditions in the low-temperature-exposure experiment conducted in this study might deviate from the wild [11]. In accordance with the cold tolerance strategy of *S*. *noctilio*, the research methods of Sinclair [20] and the description of the lower limit temperature by Lee and Denlinger [21], we tended to consider the range of SCPs as the low-temperature threshold of *S*. *noctilio*.

### Thermal tolerance as the basis for the *S*. *noctilio* global distribution prediction

The Compare Locations function in CLIMEX involves six parameters related to the temperature: DV0-lower temperature threshold, DV1-lower optimum temperature, DV2-upper optimum temperature, DV3-upper temperature threshold, TTCS-cold stress temperature threshold and TTHS-heat stress temperature threshold [8, 10]. These parameters were fitted based on our experimental data; among them, the DV1 and DV2 could not be accurately determined by this experiment.

The DV0 was set to 0 by Carnegie, Matsuki [8] based on the native distribution of *S*. *noctilio*. However, it was changed to 5°C by Ireland, Bulman [10], according to the lower thermal threshold [5.1°C] for egg-adult development [14]. The results of this recalculation are based on the wood infestation-feathering experiment conducted by Madden [12], and the minimum processing temperature was set to 12.5°C. We also indicated that larvae could survive for a long time (> 48 h) at below 5°C temperature. Therefore, we think that the DV0 might be lower than previously thought: the LLT_50_ for 48 h was -3.53°C (95% confidence interval: -6.25 to -0.41°C), the 2 h LLT_99_ was -29.77°C (95% CI: -39.79 to -26.56°C), and the SCP was -20.77 ± 0.4378°C (mean ± SEM) (range: -2.7 to -32.5°C). Therefore, the lower temperature threshold (DV0) was set to -2.7°C.

Because there have been no previous tests of the cold resistance of *S*. *noctilio*, the temperature threshold for cold stress (TTCS) was set to 0 [8] and -42°C [10]. These values are based on the native distribution of *S*. *noctilio* and the coldest location in Siberia [33]. The current setting for the TTCS is based on the cold tolerance of the species [34, 35]. Thus we set the TTCS to -32.5°C according to the lowest SCP.

We set the upper-temperature threshold (DV3) to 31°C according to the CTmax of *S*. *noctilio*. This value is very similar to the previous parameters set by Carnegie, Matsuki [8] (30°C) and Ireland, Bulman [10] (33°C). Ireland, Bulman [10] set this based on the growth limits of *S*. *noctilio* and its symbiotic fungi [12]. However, our experiments found that long-term (48 h) exposure to 33°C caused death (>30% mortality). Meanwhile, the HLT_50_ for 24 h (chi-square < 0.001) was 38.12°C (95% CI: 36.63 to 39.86°C), the HLT_50_ for 48 h was 34.78°C (95% CI: 33.96 to 35.68°C). The CTmax of larvae (37.67 ± 0.54°C) and adult (40.53 ± 0.27°C) ranged from 30.78 to 45.84°C. And the linear regression results showed values of 31.94 to 43.08°C. Furthermore, the limit of growth should be lower.

Ireland, Bulman [10] set the TTHS to 33°C because the minimum developmental time for a high mortality rate (= 60%) in the adult phase is 33.5°C [12], and 36°C to kill fungi [36]. We increased this parameter because 1) the temperature of heat stress tends to be higher than the upper limit temperature [34], and 2) our results showed that low mortality only caused on 33°C (< 40%) and is far away from the critical high temperature. Therefore, we set the TTHS to 35°C according to the long-term (48 h) high lethal temperature when the mortality was 50%.

The reasons for these modification of the existing predicted parameters were mainly based on the following points: 1) Previous studies have not considered the cold/heat tolerance of *S*. *noctilio*, the different stages and the corresponding habitat [10]. 2) There has been no previous detailed description of the thermal physiology of *S*. *noctilio* [10, 11]. 3) Madden [12] focuses on the effects of different temperatures on developmental processes, but we mainly explored the threshold of survivability temperature tolerance.

## Conclusions

We determined the tolerance temperature range of *S*.*noctilio*, measured and calculated the key inflection temperature that affects survival, and concluded that the adult was more heat-resistant and the larva was more cold-tolerant. The temperature tolerance of symbiotic fungi in domestic populations is also important for the division of the *S*. *noctilio* temperature range [37]. In addition, the tolerance of individuals significantly differed, and the influence of other factors (such as body size, environment, sampling point and sampling time) needs to be further explored [16]. With rapid climate change and rapid global trade and considering the past propagation patterns and ecological economic hazards of *S*. *noctilio*, comprehensive future research on the climate environment suitable for this species is needed to inform conservation and management initiatives.

## Supporting information

S1 File Tolerance temperature of *S. noctilio* under dynamic temperature change and extreme temperature exposure. The four tables in the document correspond to Figs 1, 2, 3 and 4.
(XLSX)
